# Inadvertent Lead Malposition in the Left Heart during Implantation of Cardiac Electric Devices: A Systematic Review

**DOI:** 10.3390/jcdd9100362

**Published:** 2022-10-20

**Authors:** Lorenzo Spighi, Francesco Notaristefano, Silvia Piraccini, Giuseppe Giuffrè, Alberto Barengo, Matteo D’Ammando, Salvatore Notaristefano, Giuseppe Bagliani, Gianluca Zingarini, Fabio Angeli, Paolo Verdecchia, Claudio Cavallini

**Affiliations:** 1 Struttura Complessa di Cardiologia, Hospital of Perugia, 06129 Perugia, Italy; 2 Department of Medicine and Surgery, University of Insubria, 21100 Varese, Italy; 3 Department of Medicine and Cardiopulmonary Rehabilitation, Maugeri Care and Research Institute, IRCCS Tradate, 21049 Tradate, Italy

**Keywords:** lead malposition, cardiac implantable electric devices, pacemaker, implantable cardioverter defibrillator, stroke, transient ischemic attack, lead extraction, anticoagulation

## Abstract

Background. The inadvertent lead malposition in the left heart (ILMLH) is an under-recognized event, which may complicate the implantation of cardiac electronic devices (CIEDs). Methods. We investigated the clinical conditions associated with ILMLH and the treatment strategies in these patients. We made a systematic review of the literature and identified 132 studies which reported 157 patients with ILMLH. Results. The mean age of patients was 68 years, and 83 were women. ILMLH was diagnosed, on average, 365 days after CIEDs implantation. Coexisting conditions were patent foramen ovale in 29% of patients, arterial puncture in 24%, perforation of the interatrial septum in 20%, atrial septal defect in 16% and perforation of the interventricular septum in 4%. At the time of diagnosis of ILMLH, 46% of patients were asymptomatic, 31% had acute TIA or stroke and 15% had overt heart failure. Overall, 14% of patients were receiving anticoagulants at the time of diagnosis of ILMLH. After diagnosis of ILMLH, percutaneous or surgical lead extraction was carried out in 93 patients (59%), whereas 43 (27%) received anticoagulation. During a mean 9-month follow-up after diagnosis of ILMLH, four patients experienced TIA or stroke (three on oral anticoagulant therapy and one after percutaneous lead extraction). Conclusion. ILMLH is a rare complication, which is usually diagnosed about one year after implantation of CIEDs. An early diagnosis of ILMLH is important. Lead extraction is a safe and effective alternative to anticoagulants.

## 1. Introduction

Inadvertent lead malposition in the left heart (ILMLH) is a rare complication, which may occur during implantation of cardiac electronic devices (CIEDs). It may be recognized either during the procedure or at a variable time distance spanning from days to years. The first case was reported in 1969 by Stillman and Richards [1], and since then only relatively few additional cases have been published.

A reliable assessment of the true prevalence of ILMLH may be prevented by the high rate of underdiagnosis, which may lead to underreporting. If the malposition is diagnosed after discharge from hospital, which may occur in up to 40% of cases, the diagnosis can be driven by a variety of clinical complications [2]. However, this condition might remain silent even for a very long time [2,3]. In a cohort of over 2000 patients receiving a CIED, ILMLH was found in 0.34% of patients [4]. ILMLH has been associated with patent foramen ovale [5], atrial or ventricular septal defect [6], perforation [1] or arterial puncture [7].

We lack specific guidelines for the management of this complication [8]. Anticoagulation with warfarin with an international normalized ratio (INR) set to >2.5 can protect from thromboembolism and subtherapeutic values during chronic therapy, which have been associated with an increased risk of stroke and TIA [9,10]. Conversely, antiplatelet therapy does not seem to be effective for the prevention of cerebrovascular events [10]. Lead extraction has been suggested as the most reasonable therapy, and it can be performed either percutaneously or surgically [3]. Since percutaneous lead extraction may carry a high risk of thrombus mobilization and embolization, it has been reserved for high surgical risk patients or those with recently implanted leads [11]. Surgical lead extraction might be the preferred strategy when leads are old or show a high thrombotic burden and when concomitant defects need surgical correction [12,13].

Because the reported data in this area remain few and sparse, we conducted the present systematic review of published cases of inadvertent lead malposition inside the left atrium or the left ventricle with the aim of investigating the diagnostic process, therapy and outcome of these patients.

## 2. Materials and Methods

This review was conducted according to the guidelines of the Preferred Reporting Items for Systematic Reviews and Meta-Analyses extension for Scoping Reviews (PRISMA-ScR) [14]. We performed a literature search using MEDLINE (through PubMed) with the keyword “lead malposition” to select published studies reporting cases of ILMLH. Studies were considered eligible if they were written in the English language and if the malposed leads were placed inside the left atrium or ventricle. We retrieved additional cases from a detailed analysis of bibliographic references cited by the selected studies and also from the Pacemaker/Implantable Cardioverter Defibrillator Registry of the University Hospital of Perugia. The latter is an observational registry of consecutive patients undergoing CIEDs surgery at the electrophysiology laboratory of the University Hospital of Perugia, approved by the local Ethical Committee.

Data about patients included in the selected reports were extracted independently by two authors (S.L and N.F) and discrepancies were resolved in conference.

The full list of extracted data is provided below.

Historical data: age at diagnosis, gender and main comorbidities;

Baseline data: time from CIEDs surgery to the diagnosis of ILMLH, indication for implantation, side of implantation, type of device implanted, mode of diagnosis of ILMLH, cause of malposition, symptoms at diagnosis and antithrombotic therapy at diagnosis;

Therapy after diagnosis of ILMLH: antithrombotic therapy, extraction type (percutaneous or surgical) and complications;

Follow-up data after diagnosis of ILMLH: length of follow-up and events at follow up (transient ischemic attack/stroke, death);

Left ventricular paced ECG data: axis on the frontal plane, polarity of the QRS in the precordial leads and DI defined as the sum of the positive and negative deflections, QRS transition in the precordial lead defined as the lead where the QRS becomes predominantly negative or isoelectric and QRS morphology (R, qR, RS, QS, Rr, rR, rS) in the precordial leads.

As for our Center, data are collected from periodical clinical visits and telephone contacts with patients.

Statistical Analysis. We used the SPSS Software, Version 22 (IBM corporation, US), for data analysis. Continuous variables are presented as mean ± standard deviation (SD) or median (interquartile range), and categorical variables as frequency (percentage). Continuous variables were compared with the Student’s *t*-test or Mann-Whitney’s test as appropriate. Categorical variables were compared through the chi-squared test or Fisher’s test as appropriate. The relationship between lead extraction and some selected explanatory variables was assessed in a multivariable analysis model by binary logistic regression. Two-sided *p* values < 0.05 were considered statistically significant.

## 3. Results

**Figure 1:**
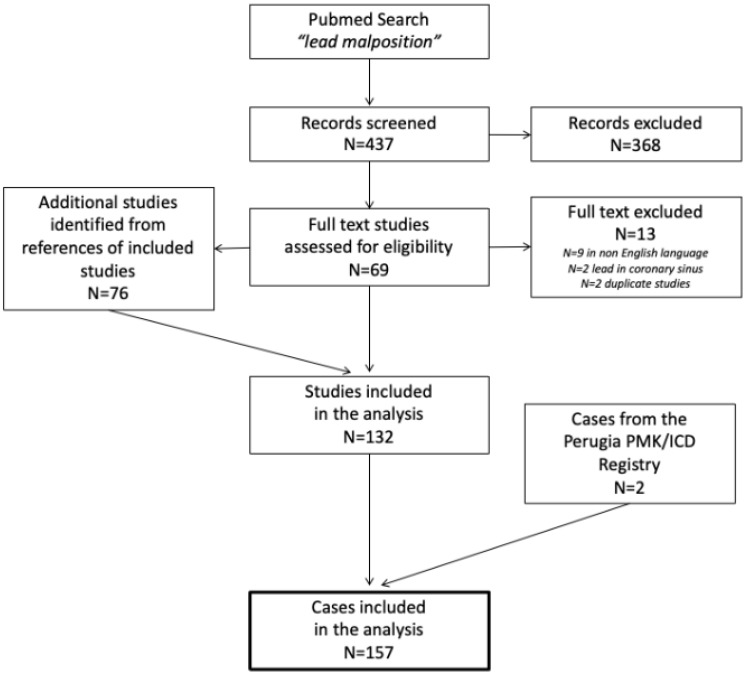
Flow diagram of the study.

Overall, 437 records were screened, 69 full text studies were assessed for eligibility, 56 of them satisfied the pre-specified review inclusion criteria and 76 additional studies were judged eligible after a detailed screening of the bibliographic references. Two additional cases were identified in the PMK/ICD Registry of the University Hospital of Perugia. Finally, 157 patients were included in the review. The flow-diagram of the study is reported in Figure 1.

Baseline characteristics. The main clinical characteristics of patients are shown in Table 1. Mean age was 68 years and 74 patients (47%) were male.

The reasons for CIEDs implantation were atrioventricular block in 41% of cases, sick sinus syndrome in 29% and primary or secondary prevention of sudden cardiac death in 11%. The median time from implantation to diagnosis of ILMLH was 365 (30–1642) days. When ILMLH was discovered, 55 patients (35%) were not taking antithrombotics, 24 patients (15%) were on antiplatelets and 22 patients (14%) were on anticoagulants. There was no association between anticoagulant therapy and TIA/stroke at presentation (*p* = 0.469).

As shown in Table 2, 46% of patients were asymptomatic at the time of ILMLH diagnosis. In the group with symptoms, 57% of patients had acute TIA or stroke (Supplementary Table S1), and 27% had acute heart failure, at the time of ILMLH diagnosis.

Malposition was confirmed through transthoracic echocardiography, chest x-ray or transesophageal echocardiography in the majority of cases. ILMLH was associated with a congenital heart disease in the vast majority of patients (patent foramen ovale in 29%, atrial septum defect in 16% and complex congenital disease in 4%) and in a minority with interatrial or interventricular septum perforation (24%) or arterial puncture (24%).

ECG. Ventricular paced ECG was available in 64 patients. As shown in Table 3, there was a right bundle branch block (RBBB) pattern in 98% of cases and 73% of cases had a QRS transition after V_3_. In 98% of cases, there was a predominantly positive QRS pattern in V_1_, and in 86%, a predominantly negative QRS pattern in Lead I. The median paced QRS axis on the frontal plane was −120° (IQR −150°–40°).

Treatment. After diagnosis of ILMLH, most patients underwent percutaneous (40%) or surgical (20%) lead extraction (Table S2) and the remaining patients were managed conservatively. In the latter group, 78% of patients received anticoagulants, 13% antiplatelets and 9% no antithrombotic therapy.

As shown in Table 4, the patients who underwent lead extraction were younger (*p* = 0.014), implanted in more recent years (*p* = 0.002) and diagnosed earlier after implantation (*p* < 0.0001), when compared with those who were treated non-invasively.

As shown in Table 5, age ≤ 75 years (OR 4.4, 95% CI 1.0–6.8, *p* = 0.001), lead dwelling time ≤ 1 year (OR 10.7, 95% CI 4.1–27.5, *p* < 0.0001) and TIA/stroke at ILMLH diagnosis (OR 2.7, 95% CI 1.0–6.8, *p* = 0.042) were independent predictors of lead extraction. Patients with congenital heart defects had the same probability of receiving surgical or percutaneous extraction (44% versus 56%, *p* = 0.265). During surgical extraction, 20 patients (64%) underwent various additional procedures including coronary artery bypass graft (N = 3), congenital defect correction (N = 8), valve repair or replacement (N = 6), epicardial PMK in (N = 5), perforation repair (N = 1) and aortic root surgery (N = 1). A procedure related sepsis was the sole reported serious complication.

Cerebral protection devices were used during percutaneous extraction in seven patients. Four procedural complications were reported during percutaneous extraction (one respiratory tract infection, one periprocedural stroke and two subclavian artery occlusion).

Follow-up. Follow-up data were available in 62 patients (39%), and the median duration of follow-up was 9 months (IQR 1–40). Follow-up duration was shorter in patients who underwent than in those who did not undergo lead extraction (2 vs. 36 months; *p* < 0.0001).

During follow-up, seven patients (11%) experienced an adverse event. Four patients developed a TIA or stroke, and three patients died. Among those with cerebrovascular event, two patients reported sub-therapeutic INR values during VKA therapy, one patient was receiving a non-Vitamin-K oral anticoagulant (NOAC) and another patient developed a stroke during percutaneous extraction. Two deaths occurred in the conservative treatment group, and one death occurred in the extraction group. Lead extraction was associated with a non-significant lower incidence of cerebrovascular events or death (6% versus 17%, *p* = 0.163).

## 4. Discussion

To the best of our knowledge, this is the largest systematic review of ILMLH. This is an important, albeit relatively rare, complication of CIEDs surgery. Ohlow et al. reported an ILMLH incidence of 0.34% in a vast cohort of patients from a tertiary center [15]. Diagnosis of ILMLH is done at a variable distance spanning days to years after the index procedure [16,17]. During lead implantation some simple maneuvers should be done routinely to avoid ILMLH. Cephalic vein cannulation virtually excludes the risk of arterial cannulation, compared to the risk carried by the subclavian or axillary approach. Fluoroscopy or ultrasound guidance may further reduce the possibility of arterial puncture. After having gained vascular access, a careful assessment of the guidewire course can confirm the cannulation of the venous system. When the implantation is from the left side, the guidewire crosses the spine from the left to the right. Independently from the implantation side, the guidewire should always be advanced below the diaphragm into the inferior vena cava. The ventricular lead can be advanced in right ventricular outflow tract before the final septal or apical position to rule out arterial or coronary sinus misplacement. Both the left anterior oblique (LAO) and the right anterior oblique (RAO) projections should be used during the implantation to guide the leads positioning. When the lead is placed in the left ventricle, the tip will cross the spine pointing towards the left ventricular lateral profile in LAO, and it will appear posterior, close to the spine in RAO; whereas, when the lead is in the left atrial appendage, the loop will appear parallel to the projection, and the tip will point towards the spine in LAO.

Careful analysis of the chest X-ray before discharge can allow identification of ILMLH in most patients, because the tip of the malposed lead is displaced more superiorly and leftward in the antero-posterior view and more posteriorly in the lateral view compared to the standard position in the right-sided chambers [18]. The definitive confirmation is usually obtained with transthoracic echocardiography. However, transesophageal echocardiography may be required to characterize associated cardiac defects and to rule out lead thrombosis.

**Figure 2:**
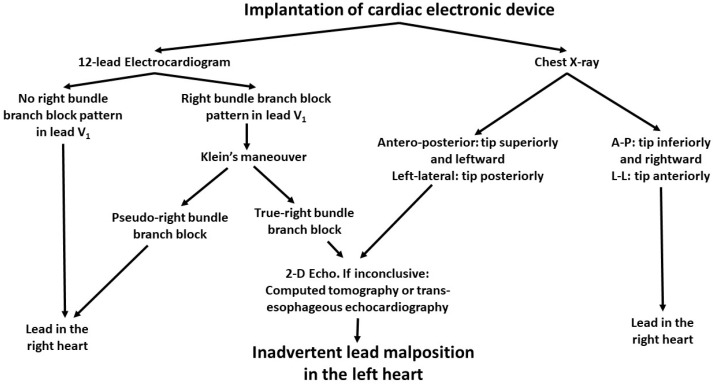
Suggested diagnostic algorithm. A right bundle branch block morphology in V1 on the ECG despite Klein’s maneuver or an atypical position on the chest X-ray should prompt further investigations to rule out lead malposition.

Twelve-lead ECG can raise suspicion of ILMLH when the QRS in V_1_ displays RBBB morphology. This pattern has been reported also in patients paced from the right ventricle, and it has been referred as “pseudo RBBB”. By lowering V_1_ and V_2_ to the fifth intercostal space, known as the Klein’s maneuver, the RBBB pattern disappears when the pacing electrode has been correctly positioned [4], whereas no changes are detected if the lead is in the left ventricle [19]. Okmen et al. developed an ECG algorithm to distinguish with high specificity and sensitivity the patients with pseudo RBBB from those with true RBBB caused by malposed leads [20]. In this study, among 12 patients with the electrode in the left ventricle, 83% had frontal axis between −30° and −90°, 100% had precordial transition after V_3_ and 100% had an absence of a S wave in DI. In our study we found the QRS axis between −30° and −90° in 82% of patients, precordial transition after V_3_ in 73% and predominantly positive QRS in DI in 87%, suggesting that Okmen’s criteria may have lower diagnostic accuracy in a larger cohort. Unfortunately Klein’s maneuver was reported only in one case, and no information can be provided about its utility [4] (Figure 2).

Congenital cardiac defects were the most common cause of ILMLH. The leads could reach left-sided chambers through a patent foramen ovale or through an unrecognized atrial or ventricular septal defect [21]. The inadvertent arterial puncture and the advancement of the lead through the aortic valve were the causes of ILMLH in 24% of patients [7,22,23,24,25], whereas atrial or ventricular septal perforation was found in the remaining patients [26,27,28].

ILMLH can remain asymptomatic and incidentally discovered, or it can present with severe symptoms. In our review, 46% of patients were fully asymptomatic when ILMLH was discovered, whereas 31% suffered a TIA/stroke, not dissimilar to the 37% reported by Van Gelder et al. [9].

Management of ILMLH remains a clinical dilemma, and guidelines do not provide solid recommendations [8]. Antiplatelet therapy does not adequately protect from thromboembolic events associated with left-sided leads, as suggested in some reports [29,30]. In the present study, the prevalence of acute TIA/stroke at the time of diagnosis did not differ between patients treated or untreated with anticoagulants (46% versus 37%%, *p* = 0.469). Anticoagulation with warfarin and INR > 2.5 has been reported to be effective in preventing thromboembolic recurrences in patients with ILMLH, but the benefit was mitigated by fluctuations in the INR, which may be associated with thromboembolism [2]. Three patients suffered a cerebrovascular ischemic event during anticoagulant therapy. Two of them had sub-therapeutic INR and one was on NOAC. The limited efficacy of Warfarin in the setting of pacing leads inside the left-sided chambers can be inferred also from studies that tested left ventricular endocardial pacing leads as part of CRT. Despite a target INR of three, the incidence of TIA/stroke was 11% in the ALSYNC trial, 7% in a more recent study and five events per 100 patient years in a meta-analysis of published studies [31,32,33]. Taken together, these data reinforced the concept of a limited utility of Warfarin in the setting of pacing leads inside the left atrium or ventricle.

Lead extraction may provide a definitive treatment, thereby avoiding the need for anticoagulation. It was performed in the majority of patients (60%). We noted that lead dwelling time less than one year, age less than 75 years and TIA/stroke at ILMLH diagnosis increased the likelihood of receiving lead extraction.

**Figure 3:**
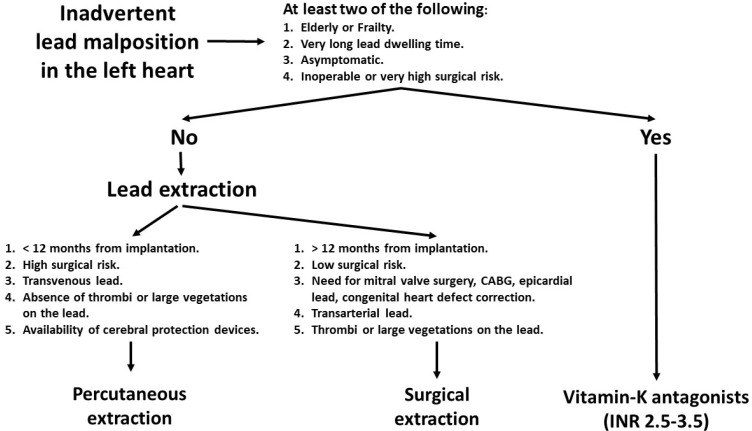
Suggested treatment algorithm. Asymptomatic patients with very high surgical risk can be managed conservatively with vitamin K antagonists and INR between 2.5 and 3.5. For patients suitable for lead extraction, the surgical procedure can be preferred if the lead dwelling time is more than one year, the embolization risk is high, the lead is placed through the arterial route and if additional interventions must be performed (e.g., epicardial leads, congenital heart defect repair).

Traditionally, surgical extraction has been considered the gold standard for removing leads inadvertently placed in the left atrium or ventricle, because it could minimize the risk of thrombi and debris dislodgement, especially when leads had a long dwelling time [3]. Moreover, the surgical procedure offered the possibility to perform additional interventions such as the repair of congenital defects, the treatment of mitral valvulopathy or the placement of epicardial electrodes [13,34]. Unexpectedly, we found that only one-third of patients in the extraction group received the surgical procedure, whereas two-thirds were treated percutaneously. Traditionally, percutaneous extraction for ILMLH has been discouraged because of the risk of thromboembolism due to lead manipulation. Indeed, the extraction tools have been designed for transvenous procedures, and the potential complications connected to their use on the arterial side are unknown. Cerebral embolic protection devices can minimize the risk of cerebral embolism through the preventive deployment of filters in the carotid and subclavian arteries [35]. However, cerebral protection was used in a minority of patients included in our overview, and one patient who experienced a periprocedural stroke had received this protective measure. A point to consider is that the low rate of complications associated with percutaneous or surgical extraction occurred in a context of devices implanted more recently. Taken together, these data suggest that percutaneous extraction might be a preferable option in older and fragile patients (Figure 3), particularly if the index procedure is quite recent, whereas surgical extraction might be preferable in younger patients with old leads. The need for epicardial pacing or presence of congenital or valvular defects, as well as transarterial leads, may also favor surgical extraction.

### Limitations

This review included data that originated from case reports and small observational series. Complications caused by extraction procedures might be under-reported, and reports on patients with favorable clinical course might have been preferentially published. The duration of follow-up differed across the studies.

## 5. Conclusions

ILMLH is a rare complication, which may occur after implantation of CIEDs. It becomes symptomatic in more than one half of patients after a variable time from the index procedure. Lead extraction, the ultimate treatment, appears to be associated with a low incidence of complications. Surgical extraction is mandatory in patients requiring additional procedures such as mitral valve or cardiac congenital defect surgery. Anticoagulation should be avoided for fragile patients and to asymptomatic patients with very old leads.

## Figures and Tables

**Table 1:** Baseline characteristics of patients with Inadvertent lead malposition in the left heart chambers.

Number of Cases		157
Age at diagnosis, years (SD)		68 (14)
Male gender, N (%)		74 (47)
Atrial Fibrillation, N (%)		23 (15)
Hypertension, N (%)		23 (15)
Ischemic Heart Disease, N (%)		23 (15)
Diabetes, N (%)		11 (7)
Mechanical Heart Valve, N (%)		2 (1)
History of Stroke/TIA, N (%)		5 (3)
Heart Failure, N (%)		14 (9)
Baseline Antithrombotic Therapy	No Antithrombotic therapy, N (%)	55 (35)
	Antiplatelets, N (%)	24 (15)
	Anticoagulants, N (%)	22 (14)
	Unknown, N (%)	56 (36)
Indication for implant	Sick Sinus Syndrome, N (%)	45 (29)
	Atrioventricular block, N (%)	64 (41)
	Primary prevention, N (%)	11 (7)
	Secondary prevention, N (%)	6 (4)
	Other, N (%)	18 (11)
	Unknown, N (%)	13 (8)
Right sided implant, N (%)		38 (24)
Type of device	Pacemaker, N (%)	138 (88)
	Implantable Cardioverter Defibrillator, N (%)	16 (10)
	Cardiac Resynchronization Therapy, N (%)	3 (2)

**Table 2:** Diagnosis of inadvertent lead malposition in the left heart chambers.

Number of Cases		157
Time to diagnosis, days (IQR)		365 (30–1642)
Symptoms at diagnosis	Asymptomatic, N (%)	73 (46)
	Transient ischemic attack or Stroke, N (%)	48 (31)
	Heart Failure, N (%)	23 (15)
	Endocarditis, N (%)	2 (1)
	Other, N (%)	11 (7)
Confirmation of Malposition	By Fluoroscopy, N (%)	3 (2)
	By Chest X-ray, N (%)	28 (18)
	By Transthoracic Echocardiography, N (%)	95 (60)
	By Transesophageal Echocardiography, N (%)	23 (15)
	By Computed Tomography, N (%)	4 (2)
	Unknown, N (%)	2 (3)
Cause of Malposition	Interatrial septum perforation, N (%)	31 (20)
	Patent foramen ovale, N (%)	46 (29)
	Atrial Septal Defect, N (%)	25 (16)
	Interventricular septum perforation, N (%)	7 (4)
	Arterial puncture, N (%)	38 (24)
	Complex congenital heart disease, N (%)	6 (4)
	Other, N (%)	4 (3)

**Table 3:** Electrocardiographic features.

QRS Transition	Number of Cases (%)
V_1_, N (%)	1 (2)
V_2_, N (%)	3 (5)
V_3_, N (%)	13 (20)
V_4_, N (%)	17 (26)
V_5_, N (%)	20 (31)
V_6_, N (%)	10 (16)
**QRS pattern in lead V_1_**	
R, N (%)	26 (40)
qR, N (%)	5 (8)
QS, N (%)	1 (2)
Rr’, N (%)	20 (31)
rR’, N (%)	12 (19)
**QRS pattern in lead L1**	
QS, N (%)	19 (30)
rS, N (%)	30 (47)
Rs, N (%)	6 (9)
R, N (%)	8 (12)
rs, N (%)	1 (2)
**QRS pattern in lead V_6_**	
QS, N (%)	19 (30)
rS, N (%)	37 (59)
R, N (%)	3 (5)
RS, N (%)	4 (6)
**QRS pattern in lead aVL**	
QS, N (%)	13 (20)
rS, N (%)	27 (42)
R, N (%)	18 (28)
qR, N (%)	1 (2)
RS, N (%)	4 (6)
**QRS pattern in lead III**	
QS, N (%)	14 (23)
rS, N (%)	11 (18)
Rr’, N (%)	2 (3)
R, N (%)	19 (31)
rR’, N (%)	2 (3)
qR, N (%)	12 (19)
RS, N (%)	2 (3)

**Table 4:** Comparison between patients treated conservatively or with lead extraction.

		Conservative Treatment *n* = 55	Lead Extraction *n* = 93	*p*-Value
Age, years (IQR)		74 (67–79)	69 (62–76)	0.014
Year of report (IQR)		2006 (1998–2011)	2011 (2003–2015)	0.002
Time from implantation, days (IQR)		875 (292–2281)	90 (2–690)	<0.0001
Male gender		23 (42%)	48 (52%)	ns
Symptoms at diagnosis	Asymptomatic, N (%)	27 (49%)	41 (44%)	ns
	TIA/Stroke, N (%)	15 (27%)	32 (34%)	0.105
	Heart Failure, N (%)	9 (16%)	12 (13%)	ns
Congenital heart disease		12 (22%)	18 (19%)	ns
Transarterial lead		12 (22%)	25 (27%)	ns
Antithrombotic therapy at diagnosis	No antithrombotics, N (%)	24 (52%)	30 (56%)	ns
	Antiplatelets, N (%)	11 (24%)	13 (24%)	ns
	Anticoagulants, N (%)	11 (24%)	11 (20%)	ns
Follow-up, months (IQR)		36 (12–72)	2 (1–6)	<0.0001

**Table 5:** Predictive factors of lead extraction. Multivariable logistic regression model.

Variable	Odds Ratio	95% Confidence Interval	*p* Value
Age ≤ 75 years old	4.4	1.0–6.8	0.001
Dwelling time ≤ 1 year	10.7	4.1–27.5	<0.0001
TIA/Stroke at ILMLH diagnosis	2.7	1.0–6.8	0.042
Congenital Heart disease	1.6	0.6–4.4	0.328
Year of the report	1.0	0.9–1.1	0.085
Male gender	1.1	0.5–2.5	0.842
Transarterial lead	1.7	0.6–4.9	0.328

## Data Availability

Data are available upon reasonable request from the corresponding author.

## Supplementary Materials

The following supporting information can be downloaded at: https://www.mdpi.com/article/10.3390/jcdd9100362/s1. Table S1: Details of patients with acute transient ischemic attack or stroke at the time of lead malposition diagnosis; Table S2: Details of patients who underwent surgical or percutaneous lead extraction [1,2,3,10,11,12,13,15,16,17,21,22,23,24,25,27,28,30,34,35,36,37,38,39,40,41,42,43,44,45,46,47,48,49,50,51,52,53,54,55,56,57,58,59,60,61,62,63,64,65,66,67,68,69,70,71,72,73,74,75,76,77,78,79,80,81,82,83,84,85,86,87,88,89,90,91,92,93,94,95,96,97,98,99,100,101,102,103,104,105,106].

## References

[1] Stillman M.T., Richards A.M. (1969). Perforation of the interventricular septum by transvenous pacemaker catheter. Diagnosis by change in pattern of depolarization on the electrocardiogram. Am. J. Cardiol..

[2] Van Gelder B.M., Bracke F.A., Oto A., Yildirir A., Haas P.C., Seger J.J., Stainback R.F., Botman K.J., Meijer A. (2000). Diagnosis and management of inadvertently placed pacing and ICD leads in the left ventricle: A multicenter experience and review of the literature. Pacing Clin. Electrophysiol..

[3] Rodriguez Y., Baltodano P., Tower A., Martinez C., Carrillo R. (2011). Management of symptomatic inadvertently placed endocardial leads in the left ventricle. Pacing Clin. Electrophysiol..

[4] Klein H.O., Beker B., Sareli P., DiSegni E., Dean H., Kaplinsky E. (1985). Unusual QRS morphology associated with transvenous pacemakers: The pseudo RBBB pattern. Chest.

[5] Shmuely H., Erdman S., Strasberg B., Rosenfeld J.B. (1992). Seven years of left ventricular pacing due to malposition of pacing electrode. Pacing Clin. Electrophysiol..

[6] Van Erckelens F., Sigmund M., Lambertz H., Kreis A., Reupcke C., Hanrath P. (1991). Asymptomatic left ventricular malposition of a transvenous pacemaker lead through a sinus venosus defect: Follow-up over 17 years. Pacing Clin. Electrophysiol..

[7] Lepore V., Pizzarelli G., Dernevik L. (1987). Inadvertent transarterial pacemaker insertion: An unusual complication. Pacing Clin. Electrophysiol..

[8] Kusumoto F.M., Schoenfeld M.H., Wilkoff B.L., Berul C.I., Birgersdotter-Green U.M., Carrillo R., Cha Y.M., Clancy J., Deharo J.C., Ellenbogen K.A. (2017). 2017 HRS expert consensus statement on cardiovascular implantable electronic device lead management and extraction. Heart Rhythm.

[9] van Gelder B.M., Scheffer M.G., Meijer A., Bracke F.A. (2007). Transseptal endocardial left ventricular pacing: An alternative technique for coronary sinus lead placement in cardiac resynchronization therapy. Heart Rhythm.

[10] Sharifi M., Sorkin R., Lakier J.B. (1994). Left heart pacing and cardioembolic stroke. Pacing Clin. Electrophysiol..

[11] Kosmidou I., Karmpaliotis D., Kandzari D.E., Dan D. (2012). Inadvertent transarterial lead placement in the left ventricle and aortic cusp: Percutaneous lead removal with carotid embolic protection and stent graft placement. Indian Pacing Electrophysiol. J..

[12] Liebold A., Aebert H., Muscholl M., Birnbaum D.E. (1994). Cerebral embolism due to left ventricular pacemaker lead: Removal with cardiopulmonary bypass. Pacing Clin. Electrophysiol..

[13] Overbeck M., Kolb C., Schmitt C., Schomig A., Lange R. (2005). Accidental transarterial implantation of dual chamber pacemaker leads in the left ventricle and the right coronary artery. Pacing Clin. Electrophysiol..

[14] Tricco A.C., Lillie E., Zarin W., O’Brien K.K., Colquhoun H., Levac D., Moher D., Peters M.D.J., Horsley T., Weeks L. (2018). PRISMA Extension for Scoping Reviews (PRISMA-ScR): Checklist and Explanation. Ann. Intern. Med..

[15] Ohlow M.A., Roos M., Lauer B., Von Korn H., Geller J.C. (2016). Incidence, predictors, and outcome of inadvertent malposition of transvenous pacing or defibrillation lead in the left heart. Europace.

[16] Schmiady M.O., Hofmann M., Maisano F., Morjan M. (2020). Do all roads lead to Rome? Treatment of malposition pacemaker lead in the left ventricle. Eur. J. Cardio-Thorac. Surg..

[17] Stouffer C.W., Shillingford M.S., Miles W.M., Conti J.B., Beaver T.M. (2010). Lead astray: Minimally invasive removal of a pacing lead in the left ventricle. Clin. Cardiol..

[18] Singh N., Madan H., Arora Y.K., Dutta R., Sofat S., Bhardwaj P., Sharma R., Chadha D.S., Ghosh A.K., Sengupta S. (2014). Malplacement of endocardial pacemaker lead in the left ventricle. Med. J. Armed Forces India.

[19] Barold S.S., Giudici M.C. (2016). Renewed interest in the significance of the tall R wave in ECG lead V1 during right ventricular pacing. Expert Rev. Med. Devices.

[20] Okmen E., Erdinler I., Oguz E., Akyol A., Turek O., Cam N., Ulufer T. (2006). An electrocardiographic algorithm for determining the location of pacemaker electrode in patients with right bundle branch block configuration during permanent ventricular pacing. Angiology.

[21] Trohman R.G., Sharma P.S. (2018). Detecting and managing device leads inadvertently placed in the left ventricle. Clevel. Clin. J. Med..

[22] Bajaj R.R., Fam N., Singh S.M. (2015). Inadvertent transarterial pacemaker lead placement. Indian Heart J..

[23] Mazzetti H., Dussaut A., Tentori C., Dussaut E., Lazzari J.O. (1990). Transarterial permanent pacing of the left ventricle. Pacing Clin. Electrophysiol..

[24] Winner S.J., Boon N.A. (1989). Transvenous pacemaker electrodes placed unintentionally in the left ventricle: Three cases. Postgrad. Med. J..

[25] Zabek A., Malecka B., Pfitzner R., Trystula M., Kruszec P., Lelakowski J. (2013). Extraction of left ventricular pacing lead inserted via the left subclavian artery. Pol. Arch. Med. Wewn..

[26] Almomani A., Abualsuod A., Paydak H., Peer W., Maskoun W. (2017). Chronic lead malposition diagnosis and management: Discussion of two cases and literature review. Clin. Case Rep..

[27] Ergun K., Tufekcioglu O., Karabal O., Ozdogan O.U., Deveci B., Golbasi Z. (2004). An unusual cause of stroke in a patient with permanent transvenous pacemaker. Jpn. Heart J..

[28] Sinha S.K., Varm C.M., Thakur R., Krishna V., Goel A., Kumar A., Jha M.J., Mishra V., Singh Syal K. (2015). An Unconventional Route of Left Ventricular Pacing. Cardiol. Res..

[29] Sharifi M., Sorkin R., Sharifi V., Lakier J.B. (1995). Inadvertent malposition of a transvenous-inserted pacing lead in the left ventricular chamber. Am. J. Cardiol..

[30] Bohm A., Banyai F., Komaromy K., Pinter A., Preda I. (1998). Cerebral embolism due to a retained pacemaker lead: A case report. Pacing Clin. Electrophysiol..

[31] Gamble J.H.P., Herring N., Ginks M., Rajappan K., Bashir Y., Betts T.R. (2018). Endocardial left ventricular pacing for cardiac resynchronization: Systematic review and meta-analysis. Europace.

[32] Geller L., Sallo Z., Molnar L., Tahin T., Ozcan E.E., Kutyifa V., Osztheimer I., Szilagyi S., Szegedi N., Abraham P. (2019). Long-term single-centre large volume experience with transseptal endocardial left ventricular lead implantation. Europace.

[33] Morgan J.M., Biffi M., Geller L., Leclercq C., Ruffa F., Tung S., Defaye P., Yang Z., Gerritse B., van Ginneken M. (2016). ALternate Site Cardiac ResYNChronization (ALSYNC): A prospective and multicentre study of left ventricular endocardial pacing for cardiac resynchronization therapy. Eur. Heart J..

[34] Ling L.F., Lever H. (2013). Six uneventful years with a pacing lead in the left ventricle. Heart Rhythm.

[35] Contractor T., Co M.L., Cooper J.M., Mandapati R., Abudayyeh I. (2020). Management of inadvertent lead placement in the left ventricle via a patent foramen ovale: A multidisciplinary approach. HeartRhythm Case Rep..

[36] Agnelli D., Ferrari A., Saltafossi D., Falcone C. (2000). Stroke Cardiembolico dovuto a malposizionamento di elettrocatetere in ventricolo sinistro. Descrizione di un caso. Ital. Heart J. Suppl..

[37] Aguilar J., Summerson C. (2002). Transarterial permanent pacing of the left ventricle. An unusual complication. Rev. Mex. Cardiol..

[38] Alan B., Dusak A., Cetincakmak M.G., Alan S. (2015). An unusual pacemaker malposition and delayed diagnosis. Dicle Med. J..

[39] Alozie A., Westphal B., Yerebakan C., Steinhoff G. (2011). Transient ischaemic attack due to the lead of an implantable defibrillator in the left heart. Interact. Cardiovasc. Thorac. Surg..

[40] Anastacio M.M., Castillo-Sang M., Lawton J.S. (2012). Laser Extraction of Pacemaker Lead Traversing a Patent Foramen Ovale and the Mitral Valve. Ann. Thorac. Surg..

[41] Arnar D.O., Kerber R.E. (2001). Cerebral Embolism Resulting from a Transvenous Pacemaker Catheter Inadvertently Placed in the Left Ventricle: A Report of Two Cases Confirmed by Echocardiography. Echocardiography.

[42] Adnan Aslam A., McIlwain E.F., Talano J.V., Ferguson T.B., McKinnie J., Kerut E.K. (1999). An Unusual Case of Embolic Stroke: A Permanent Ventricular Pacemaker Lead Entirely Within the Arterial System Documented by Transthoracic and Transesophageal Echocardiography. Echocardiogr.

[43] Bahadorani J.N., Schricker A.A., Pretorius V.G., Birgersdotter-Green U., Dominguez A., Mahmud E. (2015). Percutaneous extraction of inadvertently placed left-sided pacemaker leads with complete cerebral embolic protection. Catheter. Cardiovasc. Interv..

[44] Bauersfeld U.K., Thakur R.K., Ghani M., Yee R., Klein G.J. (1994). Malposition of transvenous pacing lead in the left ventricle: Radiographic findings. Am. J. Roentgenol..

[45] Curnis A., Bontempi L., Coppola G., Cerini M., Novo S., Dei Cas L. (2011). Undesired left ventricular pacing. G. Ital. Cardiol..

[46] Daher I.N., Schwarz E.R., Agoston I., Rahman M.A., Saeed M., Ahmad M. (2006). Live Three-Dimensional Echocardiography in Diagnosis of Interventricular Septal Perforation by Pacemaker Lead. Echocardiography.

[47] Ghani M., Thakur R.K., Boughner D., Morillo C.A., Yee R., Klein G.J. (1993). Malposition of transvenous pacing lead in the left ventricle. Pacing Clin. Electrophysiol. PACE.

[48] Feltes Guzman G.I., Vivas Balcones D., Perez de Isla L., Zamorano Gomez J.L. (2011). Long-term pacemaker lead malposition. Role of echocardiography. Rev. Esp. Cardiol. Mar..

[49] Harrison J.L., Patel R., Jogiya R., Redwood S., Rinaldi C. (2017). Use of a cerebral protection device for the laser extraction of a pacemaker lead traversing a patent foramen ovale. HeartRhythm Case Rep..

[50] Heck P.M., Hoole S.P., Cooper J.P., Begley D.A. (2012). Inadvertent placement of left ventricular endocardial pacing lead. J. Cardiovasc. Med..

[51] Hinojos A., Ilg K. (2017). Removal of Misplaced Left Ventricular Single Lead Pacemaker in a Patient Presenting with Recurrent Transient Ischemic Attacks. Spartan Med. Res. J..

[52] Kalavakunta J.K., Gupta V., Paulus B., Lapenna W. (2014). An Unusual Cause of Transient Ischemic Attack in a Patient with Pacemaker. Case Rep. Cardiol..

[53] Karavelioglu Y., Doğan T., Kalçık M., Yalçınkaya A. (2015). Malposition of an atrial pacemaker lead crossing through patent foramen ovale in a patient with ischemic stroke. Turk Kardiyol. Dern. Ars.-Arch. Turk. Soc. Cardiol..

[54] Kutarski A., Pietura R., Tomaszewski A., Czajkowski M., Boczar K. (2013). Transvenous extraction of an eight-year-old ventricular lead accidentally implanted into the left ventricle. Kardiol. Pol..

[55] McManus D.D., Mattei M.-L., Rose K., Rashkin J., Rosenthal L.S. (2009). Inadvertent Lead Placement In The Left Ventricle: A Case Report And Brief Review. Indian Pacing Electrophysiol. J..

[56] Orlov M.V., Messenger J.C., Tobias S., Smith C.W., Waider W., Winters R., Schandling A., Castellanet M. (1999). Transesophageal echocardiographic visualization of left ventricular malpositioned pacemaker electrodes: Implications for lead extraction procedures. Pacing Clin. Electrophysiol..

[57] Parikh S.S., Traub D., Wormer D., Huang D.T. (2011). Expressive aphasia in a patient with recent dual-chamber cardioverter-defibrillator implantation: A preventable complication. Cardiol. J..

[58] Raghavan C., Cashion W.R., Spencer W.H. (1996). Malposition of transvenous pacing lead in the left ventricle. Clin. Cardiol..

[59] Rath C., Andreas M., Khazen C., Wiedemann M., Habertheuer A., Kocher A. (2014). Pacemaker lead malpositioning led to subsequent ischemic strokes despite antiplatelet and anticoagulation therapy. J. Cardiothorac. Surg..

[60] Read P.A., Bowd L.M., Kalra P.R., Roberts P.R. (2005). Ventricular tachycardia and amaurosis fugax following inadvertent left ventricular pacing. Int. J. Cardiol..

[61] Reising S., Safford R., Castello R., Bosworth V., Freeman W., Kusumoto F. (2007). A Stroke of Bad Luck: Left Ventricular Pacemaker Malposition. J. Am. Soc. Echocardiogr..

[62] Roos M., Geller C., Ohlow M. (2015). Catch the Important Beats: Unmasking Inadvertently Left Ventricular Pacing. Open J. Clin. Med. Case Rep..

[63] Schiavone W.A., Castle L.W., Salcedo E., Graor R. (1984). Amaurosis Fugax in a Patient with a Left Ventricular Endocardial Pacemaker. Pacing Clin. Electrophysiol..

[64] Schon N. (2007). Inadvertently Placed Pacing Lead: A Case Report. J. Kardiol..

[65] Schulze M.R., Ostermaier R., Franke Y., Matschke K., Braun M.U., Strasser R.H. (2005). Aortic Endocarditis Caused by Inadvertent Left Ventricular Pacemaker Lead Placement. Circulation.

[66] Sivapathasuntharam D., Hyde J.A.J., Reay V., Rajkumar C. (2011). Recurrent strokes caused by a malpositioned pacemaker lead. Age Ageing.

[67] Teshome M., Ifedili I., Nayyar M., Levine Y., Holden A., Yedlapati N., Kabra R. (2020). Diagnosis and management of inadvertently placed pacemaker lead in the left ventricle following sinus venosus atrial septal defect repair surgery. HeartRhythm Case Rep..

[68] Thosani A., Raina A., Liu E., Lasorda D., Chenarides J. (2019). Malpositioned endocardial left ventricular pacing lead extraction with transcatheter cerebral embolic protection in the setting of multiple prior embolic strokes. HeartRhythm Case Rep..

[69] Al-Dashti R., Huynh T., Rosengarten M., Page P. (2002). Transvenous pacemaker malposition in the systemic circulation and pacemaker infection: A case report and review of the literature. Can. J. Cardiol..

[70] Barbarash S., Tong A. (2016). Automatic internal cardiac defibrillator lead in the left ventricle. Complex Issues Cardiovasc. Dis..

[71] Benito Martin E., Rubin Lopez J., Corros Vicente C., De La Hera Galarza J., Martin Fernandez M. (2013). Malposition of the pacemaker lead in the left ventricle. Rev. Port. Cardiol..

[72] Bodian M., Aw F., Bamba M.N., Kane A., Jobe M., Tabane A., Mbaye A., Sarr S.A., Diao M., Sarr M. (2013). Sinus venosus atrial septal defect: A rare cause of misplacement of pacemaker leads. Int. Med. Case Rep. J..

[73] Bracke F.A., Meijer A., Van Gelder L. (2004). Lead extraction for device related infections: A single-centre experience. Europace.

[74] Calvagna G.M., Patanè S., Ceresa F., Fontana A., Sicuso G., Vinci E., Muscio G., Vasquez L., Patanè F. (2015). Inadvertent implantation of a pacemaker lead in the left ventricle: A new challenge in cardiology. Int. J. Cardiol..

[75] Carrizo A., Alfie A., Amit G., Andersen G., Leguizamón J., Oseroff O. (2015). Transarterial Percutaneous Pacemaker Lead Extraction. Rev. Argent. De Cardiol..

[76] Chun J.K., Bode F., Wiegand U.K. (2004). Left ventricular malposition of pacemaker lead in Chagas’ disease. Pacing Clin. Electrophysiol. PACE.

[77] De Cock C.C., van Campen C.M., Kamp O., Visser C.A. (2003). Successful percutaneous extraction of an inadvertently placed left ventricular pacing lead. Europace.

[78] Deshmukh A., Pothineni N.V., Pant S., Paydak H. (2014). Pacemaker lead malposition: When right is not right!. Arch. Cardiovasc. Dis..

[79] Di Tommaso L., Iannelli G., Mottola M., Mannaccio V., Poli V., Esposito G., Morisco C., Vosa C. (2013). TEVAR for Iatrogenic Injury of the Distal Aortic Arch after Pacemaker Implantation. EJVES Extra.

[80] Dissmann R., Wolthoff U., Zabel M. (2013). Double left ventricular pacing following accidental malpositioning of the right ventricular electrode during implantation of a cardiac resynchronization therapy device. J. Cardiothorac. Surg..

[81] Estrada-Quintero T., Kross D.E., Gorcsan J. (1995). Identification of a malpositioned atrial pacemaker lead across a patent foramen ovale by transesophageal echocardiography. J. Am. Soc. Echocardiogr..

[82] Moorthy N., Garg N. (2013). Inadvertent temporary pacemaker lead placement in aortic sinus. Heart Views.

[83] Gondi B., Nanda N.C. (1981). Real-time, two-dimensional echocardiographic features of pacemaker perforation. Circulation.

[84] Gupta S., Annamalaisamy R., Coupe M. (2010). Misplacement of Temporary Pacing Wire into the Left Ventricle Via an Anomalous Vein. Hell. J. Cardiol. HJC Hell. Kardiol. Ep..

[85] Iliceto S., Di Biase M., Antonelli G., Favale S., Rizzon P. (1982). Two-Dimensional Echocardiographic Recognition of a Pacing Catheter Perforation of the Interventricular Septum. Pacing Clin. Electrophysiol..

[86] Irvine J.N., LaPar D.J., Mahapatra S., DiMarco J.P., Ailawadi G. (2011). Treatment of a malpositioned transcutaneous ventricular pacing lead in the left ventricle via direct aortic puncture. Europace.

[87] Judson P.L., Moore T.B., Swank M., Ashworth H.E. (1981). Two-Dimensional Echocardiograms of a Transvenous Left Ventricular Pacing Catheter. Chest.

[88] Letek A., Kurzawski J., Sadowski M. (2016). The random placement of pacing lead in the left ventricle in a patient with patent foramen ovale. Folia Cardiol..

[89] Lin J., Wang L., Zhao Y. (2017). Inadvertent left ventricular pacing and perforation by a temporary pacemaker. J. Electrocardiol..

[90] Miniard J.K. (2001). Ultrasound Diagnosis of Malpositioned Pacemaker Lead Through a Patent Foramen Ovale. J. Diagn. Med. Sonogr..

[91] Ninot S., Sánchez G., Mestres C.-A. (2003). An unusual travel of an endocardial pacing lead to the left ventricle. Interact. Cardiovasc. Thorac. Surg..

[92] Pollock J., Pollema T., Pretorius V., Birgersdotter-Green U., Cronin B. (2017). Percutaneous Laser Lead Extraction of an Inadvertently Placed Left-Sided Pacemaker Lead. J. Cardiothorac. Vasc. Anesthesia.

[93] Ross W.B., Mohiuddin S.M., Pagano T., Hughes D. (1983). Malposition of A Transvenous Cardiac Electrode Associated with Amaurosis Fugax. Pacing Clin. Electrophysiol..

[94] Rovera C., Golzio P.G., Corgnati G., Conti V., Franco E., Frea S., Moretti C. (2019). A pacemaker lead in the left ventricle: An “unexpected” finding?. J. Cardiol. Cases Dec.

[95] Ruhela M., Bagarhatta M. (2014). Right bundle branch block pacing pattern (complicated and uncomplicated) on ECG with right ventricular pacing in a single patient A case report. J. Indian Coll. Cardiol..

[96] Sahin T., Kilic T., Celikyurt U., Aygun F., Bildirici U., Agacdiken A. (2009). Asymptomatic malposition of pacemaker lead associated with thrombus. Clin. Res. Cardiol. Off. J. Ger. Card. Soc..

[97] Santarpia G., Passafaro F., Pasceri E., Mongiardo A., Curcio A., Indolfi C. (2018). Inadvertent defibrillator lead placement into the left ventricle after MitraClip implantation: A case report. Medicine.

[98] Sarubbi B., Scognamiglio G., Fusco F., Melillo E., D’Alto M., Russo M.G. (2018). A “long-standing” malpositioned pacing lead. Long-term follow-up after extraction. Monaldi Arch. Chest Dis. Arch. Monaldi Mal. Torace.

[99] Seki H., Fukui T., Shimokawa T., Manabe S., Watanabe Y., Chino K., Takanashi S. (2008). Malpositioning of a pacemaker lead to the left ventricle accompanied by posterior mitral leaflet injury. Interact. Cardiovasc. Thorac. Surg..

[100] Seethala S., Kumar A., Adhar C., Generalovich T. (2011). A Rare Cause of Cardiac Tamponade: Left Ventricular Pacemaker Malposition. Open Cardiovasc. Imaging J..

[101] Splittgerber F.H., Ulbricht L.J., Reifschneider H.-J., Probst H., Gülker H., Minale C. (1993). Left Ventricular Malposition of a Transvenous Cardioverter Defibrillator Lead: A Case Report. Pacing Clin. Electrophysiol..

[102] Syed A., Salim S., Castillo R. (2012). Incidental Finding of Malpositioned Pacing Lead in the Left Ventricle in a Patient With Subacute Subdural Hematoma. Cardiol. Res..

[103] Tobin A.M., Grodman R.S., Fisherkeller M., Nicolosi R. (1983). Two-Dimensional Echocardiographic Localization of a Malpositioned Pacing Catheter. Pacing Clin. Electrophysiol..

[104] Velankar P., Alchalabi S., Bala S., Chang S. (2014). Transarterial direct left ventricolar pacing. MDCVJ.

[105] Velibey Y., Yaylak B., Guvenc T.S., Cinier G., Kalenderoglu K., Guzelburc O., Yildirimturk O. (2018). Inadvertent Left Ventricle Endocardial or Uncomplicated Right Ventricular Pacing: How to Differentiate in the Emergency Department. J. Emerg. Med..

[106] Wynn G.J., Weston C., Cooper R.J., Somauroo J.D. (2013). Inadvertent left ventricular pacing through a patent foramen ovale: Identification, management and implications for postpacemaker implantation checks. BMJ Case Rep..

