# Supplementary material for: Inadvertent Lead Malposition in the Left Heart during Implantation of Cardiac Electric Devices: A Systematic Review
Source: J Cardiovasc Dev Dis. 2022 Oct 20;9(10):362. doi: 10.3390/jcdd9100362 (PMC9604651; doi:10.3390/jcdd9100362)
Supplement: Supplementary file 1 [file jcdd-09-00362-s001.zip › jcdd-1950336-supplementary.pdf]

Inadvertent lead malposition in the left heart during implantation of cardiac electric devices

Supplementary Appendix

Table S1. Details of patients with acute transient ischemic attack or stroke at the time of lead malposition diagnosis.

Study	Year of publication	Age	Sex	Device Type	TIA or Stroke	Antithrombotic therapy	Time from CIED implantation and diagnosis of ILMLH (days)	Baseline antithrombotic therapy details	Management	Details management	Follow up	Months of follow-up	Event
Agnelli et al [1]	2000	63	Female	PMK	Stroke	Antiplatelet	7		Percutaneous Extraction		No		
Aguilar et al [2]	2002	35	Female	PMK	TIA	None	365		Anticoagulant	INR > 2.5	Yes	72	No
Alan et al [3]	2015	71	Female	PMK	TIA	Unknown	4745		Unknown		No		
Alozie et al [4]	2012	70	Male	ICD	TIA	Antiplatelet	60		Surgical Extraction		No		
Anastacio et [5]	2012	74	Male	PMK	TIA	Anticoagulant	913	VKA	Percutaneous Extraction		No		
Arnar et al [6]	2001	69	Male	PMK	TIA	Antiplatelet	180		Anticoagulant	VKA INR > 2.5 plus Aspirin	Yes	84	No
Arnar et al [6]	2001	62	Male	PMK	Stroke	Antiplatelet	540		Surgical Extraction		Yes		No
Aslam et al [7]	1999	65	Male	PMK	Stroke	Antiplatelet	2190		Anticoagulant	VKA	Yes	8	No
Bahadorani et al [8]	2015	73	Male	PMK	Stroke	Antiplatelet	540		Percutaneous Extraction	Cerebral Protection	Yes	1	No
Bajaj et al [9]	2015	73	Male	PMK	Stroke	Antiplatelet	120		Anticoagulant	VKA INR > 2.5	No		
Bauersfeld et al [10]	1994	79	Female	PMK	Stroke	None	1095		Anticoagulant	VKA	Yes	13	No
Bohom et al [11]	1998	59	Male	PMK	Stroke	Antiplatelet	2373		Surgical Extraction		No		No
Curnis et al [12]	2011	76	Male	PMK	Stroke	Unknown	180		Percutaneous Extraction		Yes	6	No
Daher et al [13]	2006	86	Female	PMK	Stroke	Unknown	730		Anticoagulant		No		
Ergun et al [14]	2004	61	Male	PMK	Stroke	None	730		Anticoagulant	VKA	Yes	19	No
Ghani et al [15]	1993	76	Female	PMK	Stroke	None	1020		Anticoagulant	VKA	Yes	9	No
Guzman et al [16]	2011	75	Female	PMK	Stroke	None	3280		Anticoagulant	VKA	No		
Harrison et al [17]	2017	36	Female	ICD	TIA	None	4745		Percutaneous Extraction	Cerebral Protection	No		
Heck et al [18]	2010	82	Female	PMK	TIA	Anticoagulant	1460	VKA	Percutaneous Extraction		No		
Hinojos et al [19]	2017	70	Female	PMK	TIA	Anticoagulant	30	VKA	Surgical Extraction		No		
Kalavakunta et al [20]	2014	77	Male	PMK	TIA	Anticoagulant	1080	VKA with sub-therapeutic INR plus Aspirin	Anticoagulant	VKA INR > 2.5	Yes	24	No
Karavelioglu et al [21]	2016	73	Male	PMK	Stroke	Antiplatelet	690		Percutaneous Extraction		No		
Kutarski et al [22]	2011	52	Male	PMK	Stroke	None	1825		Percutaneous Extraction	Cerebral protection	Yes	0.25	Stroke intra-procedure
Liebold et al [23]	1994	56	Female	PMK	Stroke	None	1095		Surgical Extraction		No		
McManus et al [24]	2009	78	Male	PMK	Stroke	None	60		Anticoagulant	VKA INR > 2.5	No		
Ohlow et al [25]	2015	63	Female	PMK	TIA	Unknown	120		Percutaneous Extraction		No		
Ohlow et al [25]	2015	86	Female	PMK	TIA	Unknown	365		Percutaneous Extraction		No		
Orlov et al [26]	1999	27	Male	PMK	TIA	None	7		Percutaneous Extraction		No		
Orlov et al [26]	1999	77	Female	PMK	Stroke	None	7		Anticoagulant	Failed Percutaneous extraction	No		
Parikh et al [27]	2011	63	Female	ICD	Stroke	Unknown	90		Surgical Extraction		No		
Raghavan et al [28]	1996	66	Female	PMK	TIA	None	548		Surgical Extraction		No		
Rath et al [29]	2014	75	Male	PMK	Stroke	Antiplatelet	30	Antiplatelet plus LMWH	Percutaneous Extraction		No		
Read et al [30]	2005	72	Male	PMK	TIA	Anticoagulant	730	VKA with sub-therapeutic INR plus Aspirin	Surgical Extraction		Yes	4	No
Reising et al [31]	2007	80	Male	PMK	Stroke	Anticoagulant	120	VKA with sub-therapeutic INR plus Aspirin	Percutaneous Extraction		No		
Rodriguez et al [32]	2011	74	Male	ICD	TIA	Unknown	4015		Surgical Extraction		Yes	0,3	No
Roos et al [33]	2015	64	Female	PMK	TIA	Unknown	730		Percutaneous Extraction		No		
Roos et al [33]	1983	55	Male	PMK	TIA	Antiplatelet	180		Surgical Extraction		Yes		No
Schiavone et al [34]	1984	68	Female	PMK	TIA	Antiplatelet	548		Surgical Extraction		Yes	3	No
Schon et al [35]	2007	86	Female	PMK	TIA	Antiplatelet	4745		Anticoagulant	VKA INR 2-3	Yes	36	No
Schulze et al[36]	2005	82	Male	PMK	TIA	None	270		Surgical Extraction		No		
Sharifi et al [37]	1994	61	Male	PMK	TIA	Anticoagulant + Antiplatelet	1		Anticoagulant	VKA INR > 2.5	Yes	72	Stroke with sub-therapeutic INR
Sharifi et al [37]	1994	83	Female	PMK	Stroke	Antiplatelet	1825		Anticoagulant	VKA INR > 2.5	Yes	24	Stroke with sub-therapeutic INR

Sharifi et al [37]	1994	75	Female	PMK	Stroke	None	270		Anticoagulant		No		
Sivapathasuntharam et al [38]	2011	68	Male	PMK	Stroke	None	30		Surgical Extraction		No		
Teshome et al [39]	2020	46	Female	PMK	TIA	Anticoagulant	1095		Percutaneous Extraction		No		
Thosani et al [40]	2019	84	Male	PMK	TIA	Anticoagulant	1460		Percutaneous Extraction	Cerebral protection	No		
Van Gelder et al [41]	2000	75	Female	PMK	TIA	Antiplatelet	270		Surgical Extraction		No		
Winner et al [42]	1989	80	Male	PMK	Stroke	Unknown	6		Percutaneous Extraction		No		

Table S2. Details of patients who underwent surgical or percutaneous lead extraction.

First Author	Year of publication	Age	Sex	Device Type	Route	Time from CIED implantation and diagnosis of ILMLH (days)	Extraction type	Details	Procedural complication	Details complication	Follow up	Months of follow-up	Event
Agnelli et al [1]	2000	63	Female	PMK	Transvenous	7	Percutaneous extraction		No		Yes		No
Al Dashti et al [43]	2002	83	Female	PMK	Transvenous	30	Percutaneous extraction		No		No		
Alozie et al [4]	2012	70	Female	ICD	Transarterial	60	Surgical extraction		No		No		
Anastacio et al [5]	2012	74	Male	PMK	Transvenous	913	Percutaneous extraction	Laser	No		No		
Arnar et al [6]	2001	62	Male	PMK	Transvenous	540	Surgical extraction		No		Yes		No
Bahadorani et al [8]	2015	73	Male	PMK	Transarterial	540	Percutaneous extraction	Cerebral protection	No		Yes	1	No
Barbarash et al [44]	2016	63	Male	ICD	Transarterial	365	Surgical extraction		No		Yes	2	No
Benito Martin et al [45]	2013	70	Female	PMK	Transvenous	730	Surgical extraction		No		No		
Bodian et al [46]	2013	56	Female	PMK	Transvenous	1	Percutaneous extraction		No		No		
Bohm et al [11]	1998	59	Male	PMK	Transvenous	2373	Surgical extraction		No		No		
Bracke et al [47]	2003	54	Male	PMK	Transarterial	180	Percutaneous extraction	Femoral retrieval	No		Yes	6	No
Calvagna et al [48]	2015			ICD	Transarterial		Percutaneous extraction		No		No		
Carrizo et al [49]	2015	63	Female	PMK	Transarterial	90	Percutaneous extraction	Covered stent in subclavian artery	No		Yes	12	No
Case 2	2018	38	Male	PMK	Transvenous	60	Percutaneous extraction	Manual traction	No		Yes	18	No
Chun et al[50]	2004	52	Female	PMK	Transvenous	1	Percutaneous extraction		No		No		
Contractor et al [51]	2019	64	Male	CRT	Transvenous	180	Percutaneous extraction	Cerebral protection	No		Yes	1	No
Curnis et al [12]	2011	76	Male	PMK	Transvenous	180	Percutaneous extraction		No		Yes	6	No
De Cock et al [52]	2003	74	Female	PMK	Transvenous	180	Percutaneous extraction		No		No		
Deshmukh et al [53]	2014	75	Male	PMK	Transvenous	330	Percutaneous extraction		No		No		
Di Tommaso et al [54]	2013	74	Female	PMK	Transarterial	2	Percutaneous extraction	Stent graft in the aortic arch	No		Yes	12	No
Dissman et al [55]	2013	75	Male	CRT	Transvenous	30	Percutaneous extraction		No		No		
Ergun et al [14]	2004	61	Male	PMK	Transvenous	730	Percutaneous extraction		No		Yes	19	No
Ergun et al [14]	2005	63	Male	PMK	Transvenous	1	Surgical extraction	CABG	No		No		
Estrada Quintero et al [56]	1995	76	Female	PMK	Transvenous	1	Percutaneous extraction		No		No		

Garg et al [57]	2013	50	Female	PMK	Transarterial	1	Percutaneous extraction	Temporary pacing	No		No		
Gondi et al [58]	1981	58	Female	PMK	Transvenous	0.2	Percutaneous extraction	Temporary pacing	No		No		
Gupta et al [59]	2010	74	Female	PMK	Transvenous	0.2	Percutaneous extraction	Temporary pacing	No		No		
Harrison et al [17]	2017	36	Female	PMK	Transvenous	4745	Percutaneous extraction	Cerebral protection; Laser	Yes	Respiratory tract infection	No		
Heck et al [18]	2010	82	Female	PMK	Transvenous	1460	Percutaneous extraction		No		No		
Hinojos et al [19]	2017	70	Female	PMK	Transarterial	30	Surgical extraction	Epicardial PMK	No		Yes		No
Iliceto et al [60]	1982	78	Female	PMK	Transvenous	1	Percutaneous extraction		No		No		
Irvine et al [61]	2011	81	Female	PMK	Transarterial	14	Surgical extraction		No		No		
Judson et al [62]	1981	40	Male	PMK	Transvenous	2920	Surgical extraction	CABG	No		No		
Karavelioglu et al [21]	2016	73	Male	PMK	Transvenous	690	Percutaneous extraction		No		No		
Kosmidou et al [63]	2012	79	Male	PMK	Transarterial	180	Percutaneous extraction	Cerebral protection; Covered stent	No		No		
Kosmidou et al [63]	2012	63	Male	ICD	Transarterial	365	Percutaneous extraction	Cerebral protection; Covered stent	No		No		
Kumar Sinha et al [64]	2015	69	Male	PMK	Transvenous	1	Percutaneous extraction		No		Yes		No
Kutarski et al [22]	2011	52	Male	PMK	Transvenous	1825	Percutaneous extraction	Cerebral protection	Yes	Stroke peri-procedure	Yes	0.25	Stroke
Letek et al [65]	2016	74	Male	PMK	Transvenous	1	Percutaneous extraction		No		No		
Liebold et al [23]	1994	56	Female	PMK	Transarterial	1095	Surgical extraction	Epicardial PMK	No		Yes	0.25	No
Lin et al [66]	2017	82	Female	PMK	Transvenous	1	Surgical extraction	Perforation repair	No		Yes	0.5	No
Ling et al [67]	2013	61	Male	PMK	Transvenous	2190	Surgical extraction	Epicardial PMK; Aortic root surgery	No		No		
Mazzetti et al [68]	1990	64	Male	PMK	Transarterial	14	Percutaneous extraction		No		Yes	7	No
Miniard et al [69]	2001	65	Male	PMK	Transvenous	300	Percutaneous extraction		No		No		
Ninot et al [70]	2003	80	Female	PMK	Transvenous	1	Percutaneous extraction		No		No		
Ohlow et al [25]	2015	63	Female	PMK	Transarterial	120	Percutaneous extraction		No		No		
Ohlow et al [25]	2015	86	Female	PMK	Transvenous	365	Percutaneous extraction		No		No		
Ohlow et al [25]	2015	63	Male	PMK	Transvenous	0.1	Percutaneous extraction		No		No		
Ohlow et al [25]	2015	70	Female	PMK	Transvenous	1	Percutaneous extraction		No		No		
Ohlow et al [25]	2015	77	Male	PMK	Transvenous	1	Percutaneous extraction		No		No		
Ohlow et al [25]	2015	70	Male	PMK	Transvenous	1	Percutaneous extraction		No		No		
Orlov et al [26]	1999	27	Male	PMK	Transvenous	7	Percutaneous extraction		No		No		

Overbeck et al [71]	2005	72	Female	PMK	Transarterial	30	Surgical extraction	CABG	No		No		
Parikh et al [27]	2011	63	Female	ICD	Transvenous	90	Surgical extraction		No		No		
Pollock et al [72]	2016	94	Male	PMK	Transvenous	480	Percutaneous extraction		No		Yes	0.5	No
Raghavan et al [28]	1996	66	Female	PMK	Transvenous	548	Surgical extraction	ASD closure	No		No		
Rath et al [29]	2014	75	Male	PMK	Transvenous	30	Percutaneous extraction		No		No		
Read et al [30]	2005	72	Male	PMK	Transvenous	730	Surgical extraction		No		Yes	4	No
Reising et al [31]	2007	80	Male	PMK	Transarterial	120	Percutaneous extraction		No		Yes		No
Rodriguez et al [32]	2011	74	Male	ICD	Transvenous	4015	Surgical extraction	Mitral valve repair; ASD closure	No		Yes	0.3	No
Rodriguez et al [32]	2011	64	Female	CRT	Transvenous	11315	Surgical extraction	Mitral and tricuspid valve repair; ASD closure	Yes		Yes	1	Death (sepsis)
Rodriguez et al [32]	2011	74	Male	ICD	Transvenous	2555	Surgical extraction	Mitral valve replacement; ASD closure; CABG	No		Yes	1	No
Rodriguez et al [32]	2011	66	Male	PMK	Transvenous	30	Percutaneous extraction		No		Yes	3	No
Rodriguez et al [32]	2011	78	Male	PMK	Transvenous	60	Percutaneous extraction		No		Yes	6	No
Rodriguez et al [32]	2011	55	Female	PMK	Transvenous	2190	Surgical extraction		No		Yes	0.25	No
Roos et al [33]	2015	64	Female	PMK	Transvenous	730	Percutaneous extraction		No		No		
Ross et al [73]	1983	55	Male	PMK	Transvenous	180	Surgical extraction	Epicardial PMK	No		Yes		No
Rovera et al [74]	2019	68	Female	PMK	Transvenous	90	Percutaneous extraction		No		Yes	3	No
Ruhela et al [75]	2014	65	Male	PMK	Transvenous	1	Percutaneous extraction		No		No		
Sahin et al [76]	2008	54	Female	PMK	Transvenous	3285	Surgical extraction	PFO closure	No		No		
Santarpia et al [77]	2018	65	Male	ICD	Transvenous	60	Percutaneous extraction		No		Yes	1	No
Sarubbi et al [78]	2018	8	Female	PMK	Transvenous	2555	Surgical extraction	Mitral valve repair	No		Yes	140	No
Schiavone et al [34]	1984	68	Female	PMK	Transvenous	548	Surgical extraction	Epicardial PMK	No		Yes	3	No
Schmiady et al [79]	2019	80	Female	PMK	Transarterial	1	Surgical extraction		No		No		
Schulze et al [36]	2005	82	Male	PMK	Transarterial	270	Surgical extraction	Aortic valve replacement	No		No		
Seki et al [80]	2009	70	Female	PMK	Transvenous	1825	Surgical extraction	Mitral valve repair; ASD closure; epicardial PMK	No		No		
Sheetala et al [81]	2011	81	Female	PMK	Transvenous	0.2	Percutaneous extraction		No		No		
Sivapathasuntharam et al [38]	2011	68	Male	PMK	Transarterial	30	Surgical extraction		No		No		
Spilttgerber et al [82]	1993	51	Male	ICD	Transvenous	14	Surgical extraction	PFO closure	No		No		
Stillman et al [83]	1969	64	Male	PMK	Transvenous	0.2	Percutaneous extraction		No		No		

Stouffer et al [84]	2009	32	Female	PMK	Transvenous	5475	Surgical extraction	PFO closure	No		Yes	0.2	No
Syed et al [85]	2012	89	Female	PMK	Transvenous	60	Percutaneous extraction		No		No		
Teshome et al [39]	2020	46	Female	PMK	Transvenous	1095	Percutaneous extraction	Laser	No		No		
Thosani et al [40]	2019	84	Male	PMK	Transarterial	1460	Percutaneous extraction	Cerebral protection	No		No		
Tobin et al [86]	1983	83	Female	PMK	Transvenous	0.2	Percutaneous extraction		No		No		
Trohman et al [87]	1991	62	Female	PMK	Transvenous	300	Percutaneous extraction		No		No		
Van Gelder et al [41]	2000	75	Female	PMK	Transvenous	270	Surgical extraction	ASD closure; epicardial PMK	No		No		
Velankar et al [88]	2014	77	Male	PMK	Transarterial		Percutaneous extraction		No		No		
Velibey et al [89]	2018	65	Female	PMK	Transvenous	0.01	Percutaneous extraction		No		No		
Winner et al [42]	1989	70	Female	PMK	Transarterial	5	Percutaneous extraction		Yes	Subclavian artery occlusion	No		
Winner et al [42]	1989	80	Male	PMK	Transarterial	6	Percutaneous extraction		Yes	Subclavian artery occlusion	No		
Wynn et al [90]	2013	81	Mlale	PMK	Transvenous	30	Percutaneous extraction		No		Yes		No
Zabek et al [91]	2013	90	Female	PMK	Transarterial	42	Percutaneous extraction		No		Yes		No

Supplementary references

1. Agnelli D, Ferrari A, Saltafossi D, Falcone C. Stroke Cardiembolico dovuto a malposizionamento di elettrocatetere in ventricolo sinistro. Descrizione di un caso. . Ital Heart J Suppl 2000;1:122-125.
2. Aguilar J, Summerson C. Transarterial permanent pacing of the left ventricle. An unusual complication. Rev Mex Cardiol 2002;13:56-58.
3. Alan B, Susak A, Cetincakmak M, Alan S. An unusual pacemaker malposition and delayed diagnosis. Dicle Medical Journal 2015;42:253-255.
4. Alozie A, Westphal B, Yerebakan C, Steinhoff G. Transient ischaemic attack due to the lead of an implantable defibrillator in the left heart. Interact Cardiovasc Thorac Surg Jan 2012;14:128-130.
5. Anastacio MM, Castillo-Sang M, Lawton JS. Laser extraction of pacemaker lead traversing a patent foramen ovale and the mitral valve. Ann Thorac Surg Dec 2012;94:2125-2127.
6. Arnar DO, Kerber RE. Cerebral embolism resulting from a transvenous pacemaker catheter inadvertently placed in the left ventricle: a report of two cases confirmed by echocardiography. Echocardiography Nov 2001;18:681-684.
7. Adnan Aslam A, McIlwain EF, Talano JV, Ferguson TB, McKinnie J, Kerut EK. An Unusual Case of Embolic Stroke: A Permanent Ventricular Pacemaker Lead Entirely Within the Arterial System Documented by Transthoracic and Transesophageal Echocardiography. Echocardiography May 1999;16:373-378.
8. Bahadorani JN, Schricker AA, Pretorius VG, Birgersdotter-Green U, Dominguez A, Mahmud E. Percutaneous extraction of inadvertently placed left-sided pacemaker leads with complete cerebral embolic protection. Catheter Cardiovasc Interv Oct 2015;86:777-785.
9. Bajaj RR, Fam N, Singh SM. Inadvertent transarterial pacemaker lead placement. Indian Heart J Sep-Oct 2015;67:452-454.
10. Bauersfeld UK, Thakur RK, Ghani M, Yee R, Klein GJ. Malposition of transvenous pacing lead in the left ventricle: radiographic findings. AJR Am J Roentgenol Feb 1994;162:290-292.
11. Bohm A, Banyai F, Komaromy K, Pinter A, Preda I. Cerebral embolism due to a retained pacemaker lead: a case report. Pacing and clinical electrophysiology : PACE Mar 1998;21:629-630.
12. Curnis A, Bontempi L, Coppola G, Cerini M, Novo S, Dei Cas L. [Undesired left ventricular pacing]. Giornale italiano di cardiologia Nov 2011;12:724-725.
13. Daher IN, Saeed M, Schwarz ER, Agoston I, Rahman MA, Ahmad M. Live three-dimensional echocardiography in diagnosis of interventricular septal perforation by pacemaker lead. Echocardiography May 2006;23:428-429.
14. Ergun K, Tufekcioglu O, Karabal O, Ozdogan OU, Deveci B, Golbasi Z. An unusual cause of stroke in a patient with permanent transvenous pacemaker. Jpn Heart J Sep 2004;45:873-875.
15. Ghani M, Thakur RK, Boughner D, Morillo CA, Yee R, Klein GJ. Malposition of transvenous pacing lead in the left ventricle. Pacing and clinical electrophysiology : PACE Sep 1993;16:1800-1807.
16. Feltes Guzman GI, Vivas Balcones D, Perez de Isla L, Zamorano Gomez JL. Long-term pacemaker lead malposition. Role of echocardiography. Rev Esp Cardiol Mar 2011;64:250.
17. Harrison JL, Patel R, Jogiya R, Redwood S, Rinaldi CA. Use of a cerebral protection device for the laser extraction of a pacemaker lead traversing a patent foramen ovale. HeartRhythm case reports Oct 2017;3:447-449.

18. Heck PM, Hoole SP, Cooper JP, Begley DA. Inadvertent placement of left ventricular endocardial pacing lead. Journal of cardiovascular medicine Oct 2012;13:656-659.
19. Hinojos A, Ilg K. Removal of Misplaced Left Ventricular Single Lead Pacemaker in a Patient Presenting with Recurrent Transient Ischemic Attacks. Spartan Med Res J Aug 24 2017;2:6068.
20. Kalavakunta JK, Gupta V, Paulus B, Lapenna W. An unusual cause of transient ischemic attack in a patient with pacemaker. Case Rep Cardiol 2014;2014:265759.
21. Karavelioglu Y, Dogan T, Kalcik M, Yalcinkaya A. Malposition of an atrial pacemaker lead crossing through patent foramen ovale in a patient with ischemic stroke. Turk Kardiyol Dern Ars Jan 2016;44:87.
22. Kutarski A, Pietura R, Tomaszewski A, Czajkowski M, Boczar K. Transvenous extraction of an eight-year-old ventricular lead accidentally implanted into the left ventricle. Kardiologia polska 2013;71:1317-1321.
23. Liebold A, Aebert H, Muscholl M, Birnbaum DE. Cerebral embolism due to left ventricular pacemaker lead: removal with cardiopulmonary bypass. Pacing and clinical electrophysiology : PACE Dec 1994;17:2353-2355.
24. McManus DD, Mattei ML, Rose K, Rashkin J, Rosenthal LS. Inadvertent lead placement in the left ventricle: a case report and brief review. Indian pacing and electrophysiology journal Jul 1 2009;9:224-228.
25. Ohlow MA, Roos M, Lauer B, Von Korn H, Geller JC. Incidence, predictors, and outcome of inadvertent malposition of transvenous pacing or defibrillation lead in the left heart. Europace Jul 2016;18:1049-1054.
26. Orlov MV, Messenger JC, Tobias S, Smith CW, Waider W, Winters R, Schandling A, Castellanet M. Transesophageal echocardiographic visualization of left ventricular malpositioned pacemaker electrodes: implications for lead extraction procedures. Pacing and clinical electrophysiology : PACE Sep 1999;22:1407-1409.
27. Parikh SS, Traub D, Wormer D, Huang DT. Expressive aphasia in a patient with recent dual-chamber cardioverter-defibrillator implantation: a preventable complication. Cardiol J 2011;18:197-199.
28. Raghavan C, Cashion WR, Jr., Spencer WH, 3rd. Malposition of transvenous pacing lead in the left ventricle. Clin Cardiol Apr 1996;19:335-338.
29. Rath C, Andreas M, Khazen C, Wiedemann D, Habertheuer A, Kocher A. Pacemaker lead malpositioning led to subsequent ischemic strokes despite antiplatelet and anticoagulation therapy. J Cardiothorac Surg Mar 20 2014;9:54.
30. Read PA, Bowd LM, Kalra PR, Roberts PR. Ventricular tachycardia and amaurosis fugax following inadvertent left ventricular pacing. Int J Cardiol Mar 30 2005;99:479-480.
31. Reising S, Safford R, Castello R, Bosworth V, Freeman W, Kusumoto F. A stroke of bad luck: left ventricular pacemaker malposition. J Am Soc Echocardiogr Nov 2007;20:1316 e1311-1313.
32. Rodriguez Y, Baltodano P, Tower A, Martinez C, Carrillo R. Management of symptomatic inadvertently placed endocardial leads in the left ventricle. Pacing and clinical electrophysiology : PACE Oct 2011;34:1192-1200.
33. Roos M, Geller C, Ohlow M. Catch the Important Beats: Unmasking Inadvertently Left Ventricular Pacing. Open J Clin Med Case Rep 2015;1:1-4.
34. Schiavone WA, Castle LW, Salcedo E, Graor R. Amaurosis fugax in a patient with a left ventricular endocardial pacemaker. Pacing and clinical electrophysiology : PACE Mar 1984;7:288-292.
35. Schon N. Inadvertently Placed Pacing Lead: A Case Report. J Kardiol 2007;14:228-230.

36. Schulze MR, Ostermaier R, Franke Y, Matschke K, Braun MU, Strasser RH. Images in cardiovascular medicine. Aortic endocarditis caused by inadvertent left ventricular pacemaker lead placement. Circulation Dec 20 2005;112:e361-363.
37. Sharifi M, Sorkin R, Lakier JB. Left heart pacing and cardioembolic stroke. Pacing and clinical electrophysiology : PACE Oct 1994;17:1691-1696.
38. Sivapathasuntharam D, Hyde JA, Reay V, Rajkumar C. Recurrent strokes caused by a malpositioned pacemaker lead. Age Ageing May 2012;41:420-421.
39. Teshome M, Ifedili I, Nayyar M, Levine Y, Holden A, Yedlapati N, Kabra R. Diagnosis and management of inadvertently placed pacemaker lead in the left ventricle following sinus venosus atrial septal defect repair surgery. HeartRhythm case reports May 2020;6:279-282.
40. Thosani A, Raina A, Liu E, Lasorda D, Chenarides J. Malpositioned endocardial left ventricular pacing lead extraction with transcatheter cerebral embolic protection in the setting of multiple prior embolic strokes. HeartRhythm case reports Nov 2019;5:552-554.
41. Van Gelder BM, Bracke FA, Oto A, Yildirir A, Haas PC, Seger JJ, Stainback RF, Botman KJ, Meijer A. Diagnosis and management of inadvertently placed pacing and ICD leads in the left ventricle: a multicenter experience and review of the literature. Pacing and clinical electrophysiology : PACE May 2000;23:877-883.
42. Winner SJ, Boon NA. Transvenous pacemaker electrodes placed unintentionally in the left ventricle: three cases. Postgrad Med J Feb 1989;65:98-102.
43. R AL-D, Huynh T, Rosengarten M, Page P. Transvenous pacemaker malposition in the systemic circulation and pacemaker infection: a case report and review of the literature. Can J Cardiol Aug 2002;18:887-890.
44. Barbarash S, Tong A. Automatic internal cardiac defibrillator lead in the left ventricle. Complex Issues of Cardiovascular Diseases 2016;2:111-114.
45. Benito Martin E, Rubin Lopez J, Corros Vicente C, De La Hera Galarza J, Martin Fernandez M. Malposition of the pacemaker lead in the left ventricle. Rev Port Cardiol 2013;32:633-635.
46. Bodian M, Aw F, Bamba MN, Kane A, Jobe M, Tabane A, Mbaye A, Sarr SA, Diao M, Sarr M, Ba SA. Sinus venosus atrial septal defect: a rare cause of misplacement of pacemaker leads. Int Med Case Rep J 2013;6:29-32.
47. Bracke FA, Meijer A, van Gelder LM. Lead extraction for device related infections: a single-centre experience. Europace May 2004;6:243-247.
48. Calvagna GM, Patane S, Ceresa F, Fontana A, Sicuso G, Vinci E, Muscio G, Vasquez L, Patane F. Inadvertent implantation of a pacemaker lead in the left ventricle: A new challenge in cardiology. Int J Cardiol Jan 1 2016;202:914-917.
49. Carrizo A, Alfie A, Amit G, Andersen G, Leguizamón J, Oseroff O. Transarterial Percutaneous Pacemaker Lead Extraction. Revista Argentina de Cardiología 2015;83:443-444.
50. Chun JK, Bode F, Wiegand UK. Left ventricular malposition of pacemaker lead in Chagas' disease. Pacing and clinical electrophysiology : PACE Dec 2004;27:1682-1685.
51. Contractor T, Co ML, Cooper JM, Mandapati R, Abudayyeh I. Management of inadvertent lead placement in the left ventricle via a patent foramen ovale: A multidisciplinary approach. HeartRhythm case reports Feb 2020;6:89-93.
52. de Cock CC, van Campen CM, Kamp O, Visser CA. Successful percutaneous extraction of an inadvertently placed left ventricular pacing lead. Europace Apr 2003;5:195-197.
53. Deshmukh A, Pothineni NV, Pant S, Paydak H. Pacemaker lead malposition: When right is not right! Archives of cardiovascular diseases Mar 2014;107:201-202.

54. Di Tommaso L, Iannelli G, Mottola M, Mannaccio V, Poli V, Esposito G, Morisco C, Vosa C. TEVAR for Iatrogenic Injury of the Distal Aortic Arch after Pacemaker Implantation. EJVES extra 2013;26:e17-e19.
55. Dissmann R, Wolthoff U, Zabel M. Double left ventricular pacing following accidental malpositioning of the right ventricular electrode during implantation of a cardiac resynchronization therapy device. J Cardiothorac Surg Jun 27 2013;8:162.
56. Estrada-Quintero T, Kross DE, Gorcsan J, 3rd. Identification of a malpositioned atrial pacemaker lead across a patent foramen ovale by transesophageal echocardiography. J Am Soc Echocardiogr Jul-Aug 1995;8:560-562.
57. Garg N, Moorthy N. Inadvertent temporary pacemaker lead placement in aortic sinus. Heart Views Oct 2013;14:182-184.
58. Gondi B, Nanda NC. Real-time, two-dimensional echocardiographic features of pacemaker perforation. Circulation Jul 1981;64:97-106.
59. Gupta S, Annamalaisamy R, Coupe M. Misplacement of Temporary Pacing Wire into the Left Ventricle Via an Anomalous Vein. Hellenic journal of cardiology : HJC = Hellenike kardiologike epitheorese 2010;51:175-177.
60. Iliceto S, Di Biase M, Antonelli G, Favale S, Rizzon P. Two-dimensional echocardiographic recognition of a pacing catheter perforation of the interventricular septum. Pacing and clinical electrophysiology : PACE Nov 1982;5:934-936.
61. Irvine JN, LaPar DJ, Mahapatra S, DiMarco JP, Ailawadi G. Treatment of a malpositioned transcutaneous ventricular pacing lead in the left ventricle via direct aortic puncture. Europace Aug 2011;13:1207-1208.
62. Judson PL, Moore TB, Swank M, Ashworth HE. Two-dimensional echocardiograms of a transvenous left ventricular pacing catheter. Chest Aug 1981;80:228-230.
63. Kosmidou I, Karmpaliotis D, Kandzari DE, Dan D. Inadvertent transarterial lead placement in the left ventricle and aortic cusp: percutaneous lead removal with carotid embolic protection and stent graft placement. Indian pacing and electrophysiology journal Nov 2012;12:269-273.
64. Sinha SK, Varm CM, Thakur R, Krishna V, Goel A, Kumar A, Jha MJ, Mishra V, Singh Syal K. An Unconventional Route of Left Ventricular Pacing. Cardiol Res Oct 2015;6:324-328.
65. Letek A, Kurzawski J, Sadowski M. The random placement of pacing lead in the left ventricle in a patient with patent foramen ovale. Folia Cardiologica 2016;11:535-538.
66. Lin J, Wang L, Zhao Y. Inadvertent left ventricular pacing and perforation by a temporary pacemaker. Journal of electrocardiology Sep - Oct 2017;50:686-689.
67. Ling LF, Lever H. Six uneventful years with a pacing lead in the left ventricle. Heart Rhythm Apr 2013;10:614-615.
68. Mazzetti H, Dussaut A, Tentori C, Dussaut E, Lazzari JO. Transarterial permanent pacing of the left ventricle. Pacing and clinical electrophysiology : PACE May 1990;13:588-592.
69. Miniard J. Ultrasound Diagnosis of Malpositioned Pacemaker Lead Through a Patent Foramen Ovale. JDMS 2001;17:172-174.
70. Ninot S, Sanchez G, Mestres CA. An unusual travel of an endocardial pacing lead to the left ventricle. Interact Cardiovasc Thorac Surg Dec 2003;2:624-625.
71. Overbeck M, Kolb C, Schmitt C, Schomig A, Lange R. Accidental transarterial implantation of dual chamber pacemaker leads in the left ventricle and the right coronary artery. Pacing and clinical electrophysiology : PACE May 2005;28:469-471.
72. Pollock J, Pollema T, Pretorius V, Birgersdotter-Green U, Cronin B. Percutaneous Laser Lead Extraction of an Inadvertently Placed Left-Sided Pacemaker Lead. J Cardiothorac Vasc Anesth Apr 2017;31:663-668.

73. Ross WB, Mohiuddin SM, Pagano T, Hughes D. Malposition of a transvenous cardiac electrode associated with amaurosis fugax. Pacing and clinical electrophysiology : PACE Jan 1983;6:119-124.
74. Rovera C, Golzio PG, Corgnati G, Conti V, Franco E, Frea S, Moretti C. A pacemaker lead in the left ventricle: An "unexpected" finding? J Cardiol Cases Dec 2019;20:228-231.
75. Ruhela M, Bagarhatta M. Right bundle branch block pacing pattern (complicated and uncomplicated) on ECG with right ventricular pacing in a single patient A case report. Journal of Indian College of Cardiology 2014;4:31-35.
76. Sahin T, Kilic T, Celikyurt U, Aygun F, Bildirici U, Agacdiken A. Asymptomatic malposition of pacemaker lead associated with thrombus. Clinical research in cardiology : official journal of the German Cardiac Society Jan 2009;98:71-73.
77. Santarpia G, Passafaro F, Pasceri E, Mongiardo A, Curcio A, Indolfi C. Inadvertent defibrillator lead placement into the left ventricle after MitraClip implantation: A case report. Medicine (Baltimore) May 2018;97:e0733.
78. Sarubbi B, Scognamiglio G, Fusco F, Melillo E, D'Alto M, Russo MG. A "long-standing" malpositioned pacing lead. Long-term follow-up after extraction. Monaldi archives for chest disease = Archivio Monaldi per le malattie del torace Sep 4 2018;88:927.
79. Schmiady MO, Hofmann M, Maisano F, Morjan M. Do all roads lead to Rome? Treatment of malposition pacemaker lead in the left ventricle. Eur J Cardiothorac Surg May 1 2020;57:1009-1010.
80. Seki H, Fukui T, Shimokawa T, Manabe S, Watanabe Y, Chino K, Takanashi S. Malpositioning of a pacemaker lead to the left ventricle accompanied by posterior mitral leaflet injury. Interact Cardiovasc Thorac Surg Feb 2009;8:235-237.
81. Seethala S, Kumar A, Adhar C, Generalovich T. A Rare Cause of Cardiac Tamponade: Left Ventricular Pacemaker Malposition. The Open Cardiovascular Imaging Journal 2011;3:1-3.
82. Splittgerber FH, Ulbricht LJ, Reifschneider HJ, Probst H, Gulker H, Minale C. Left ventricular malposition of a transvenous cardioverter defibrillator lead: a case report. Pacing and clinical electrophysiology : PACE May 1993;16:1066-1069.
83. Stillman MT, Richards AM. Perforation of the interventricular septum by transvenous pacemaker catheter. Diagnosis by change in pattern of depolarization on the electrocardiogram. Am J Cardiol Aug 1969;24:269-273.
84. Stouffer CW, Shillingford MS, Miles WM, Conti JB, Beaver TM. Lead astray: minimally invasive removal of a pacing lead in the left ventricle. Clin Cardiol Jun 2010;33:E109-110.
85. Syed A, Salim S, Castillo R. Incidental Finding of Malpositioned Pacing Lead in the Left Ventricle in a Patient With Subacute Subdural Hematoma. Cardiol Res Aug 2012;3:187-188.
86. Tobin AM, Grodman RS, Fisherkeller M, Nicolosi R. Two-dimensional echocardiographic localization of a malpositioned pacing catheter. Pacing and clinical electrophysiology : PACE Mar 1983;6:291-299.
87. Trohman RG, Sharma PS. Detecting and managing device leads inadvertently placed in the left ventricle. Cleveland Clinic journal of medicine Jan 2018;85:69-75.
88. Velankar P, Alchalabi S, Bala S, Chang S. Transarterial direct left ventricolar pacing. MDCVJ 2014;10:255-256.
89. Velibey Y, Yaylak B, Guvenc TS, Cinier G, Kalenderoglu K, Guzelburc O, Yildirimturk O. Inadvertent Left Ventricle Endocardial or Uncomplicated Right Ventricular Pacing: How to Differentiate in the Emergency Department. The Journal of emergency medicine May 2018;54:e91-e95.

90. Wynn GJ, Weston C, Cooper RJ, Somauroo JD. Inadvertent left ventricular pacing through a patent foramen ovale: identification, management and implications for postpacemaker implantation checks. BMJ Case Rep Jun 27 2013;2013.

91. Zabek A, Malecka B, Pfitzner R, Trystula M, Kruszec P, Lelakowski J. Extraction of left ventricular pacing lead inserted via the left subclavian artery. Pol Arch Med Wewn 2013;123:560-561.
